# CRISPR/Cas9-Mediated Development of Potato Varieties with Long-Term Cold Storage and Bruising Resistance

**DOI:** 10.3390/biology14040445

**Published:** 2025-04-20

**Authors:** Gabriela Alejandra Massa, Cecilia Andrea Décima Oneto, Matías Nicolás González, Anabela Poulsen Hornum, Ailín Arizmendi, Sofía Sucar, Silvina Beatriz Divito, Sergio Enrique Feingold

**Affiliations:** 1 Laboratorio de Agrobiotecnología, EEA Balcarce-IPADS (UEDD INTA–CONICET), Instituto Nacional de Tecnología Agropecuaria (INTA), Balcarce B7620, Argentina; cdecimaoneto001@dundee.ac.uk (C.A.D.O.); matias.gonzalez@slu.se (M.N.G.); poulsenhornum.a@inta.gob.ar (A.P.H.); arizmendi.ailin@inta.gob.ar (A.A.); sofiasucar@yahoo.com.ar (S.S.); divito.silvina@inta.gob.ar (S.B.D.); 2 Facultad de Ciencias Agrarias, Universidad Nacional de Mar del Plata, Balcarce B7620, Argentina

**Keywords:** *Solanum tuberosum*, genome editing, vacuolar invertase, cold-induced sweetening, enzymatic browning, polyphenol oxidase, multi-target, ribonucleoprotein

## Abstract

Enzymatic browning and cold-induced sweetening (CIS) affect the post-harvest quality of potato tubers. Browning is caused by polyphenol oxidase 2 (PPO2), which is activated by mechanical damage during harvest and storage. CIS occurs when vacuolar invertase converts sucrose into reducing sugars, which react with amino acids during frying, forming brown pigments and acrylamide. While cold storage prevents sprouting and disease, it also increases vacuolar invertase expression, leading to quality loss. Using CRISPR/Cas9, we developed gene-edited potato lines with improved resistance to browning and CIS. Line 6A (cv. Atlantic) and E03-3 (cv. Spunta) exhibited complete vacuolar invertase (*InvVac*) knockout, maintaining chip quality for at least 60 days at 4 °C. Line 6A, renamed PIRU INTA, was tested in field trials and preserved frying quality for up to 90 days under cold storage. PIRU INTA is currently undergoing registration as a new variety. Additionally, lines E04-5B and E03-3 (cv. Spunta) showed partial *PPO2* gene edits, reducing enzymatic browning by 80% and 40%, respectively. This study demonstrates the potential of CRISPR/Cas9 to develop non-transgenic, gene-edited potatoes with enhanced storage quality, benefiting both growers and the food industry.

## Simple Summary

Potatoes can be easily damaged during harvesting and handling, leading to dark spots and quality loss. Storing them in cold temperatures helps prevent sprouting and diseases, but it can also cause an increase in sugars, making them less suitable for cooking and processing. Using gene editing, we have developed new non-GMO potato varieties that resist browning when cut or bruised and can be stored at low temperatures for at least 120 days without losing their quality. These improvements help reduce food waste and offer better storage options for both consumers and the food industry.

## 1. Introduction

Potato (*Solanum tuberosum* L.) is the third most important crop for human consumption and the fourth in terms of production worldwide, with around 350 million tons produced yearly [1]. Of the total production, around half is destined for fresh human consumption, while most of the rest is used in the preparation of processed food products, animal feed, and seed production [2].

Enzymatic browning and cold-induced sweetening affect the post-harvest quality of potato tubers. Tuber browning primarily results from the action of polyphenol oxidase 2 (PPO2), which is activated when mechanical damage during harvest, transportation, or storage disrupts cellular integrity, releasing PPO2 from vacuoles into the cytoplasm, where it encounters its phenolic substrates, later oxidized to quinones. These quinones then react with amino acids or free radicals in proteins, leading to the formation of dark-colored precipitates (Mayer, 2006) [3]. Cold storage prevents sprouting and minimizes diseases, but it also increases vacuolar invertase expression [4]. Cold-induced sweetening occurs as a response to abiotic stress, where vacuolar invertase hydrolyzes sucrose into the reducing sugars fructose and glucose. The accumulation of these sugars is problematic for industrial potato chip and french fries production, as high frying temperatures lead to the formation of dark brown polymeric pigments and harmful compounds, such as acrylamide [5,6].

Potato improvement through conventional breeding is a laborious and time-consuming process due to its tetraploid nature, high level of heterozygosity, and narrow genetic base. As a clonal crop, incremental breeding on existing successful varieties cannot be achieved through backcrossing, as in autogamous or hybrid crops, unless biotechnological techniques such as genetic transformation or gene editing are applied [7,8]. Our group has previously developed a variety derived from cv. Desirée, edited in all four alleles of the PPO2 gene, resulting in reductions of up to 69% in PPO enzymatic activity and 73% in tuber enzymatic browning [9,10]. Previous studies have demonstrated that the loss of function of the vacuolar invertase gene through gene editing is sufficient to produce potatoes that maintain chip quality during cold storage [11,12]. Additionally, the simultaneous editing of the vacuolar invertase and asparagine synthetase genes in cvs. Atlantic and Desirée using stable transformation with the CRISPR/Cas9 system has been reported to reduce acrylamide concentration by up to 80% [13]. Therefore, applying gene editing techniques to address enzymatic browning and cold-induced sweetening simultaneously result in the development of potato varieties with superior post-harvest quality.

This study aims to develop new potato varieties lacking functional vacuolar invertase through transient CRISPR/Cas9 expression in cvs. Atlantic and Spunta. Furthermore, we report the application of this technology to interrupting the vacuolar invertase and polyphenol oxidase 2 genes simultaneously in the Spunta variety, leading to improved lines with reductions in both cold-induced sweetening and enzymatic browning. Our findings highlight the effectiveness of non-transgenic gene editing in producing potato varieties with enhanced post-harvest traits, including improved cold storage performance and reduced susceptibility to bruising.

## 2. Materials and Methods

### 2.1. sgRNA Design for the Vacuolar Invertase and Polyphenol Oxidase 2 Genes of Solanum tuberosum cv. Atlantic and cv. Spunta

#### 2.1.1. Vacuolar Invertase Gene (InvVac)

The reference sequence PGSC0003DMG400013856 [14] was used for the primer design for the amplification of the *InvVac* gene in *Solanum tubersoum* cv. Atlantic and cv. Spunta. Primers InvVac-F1 and InvVac-R5 (Table 1) were used to amplify a fragment of 2874 bp from the 5’end of the target gene, using 10 ng of genomic DNA as a template in a reaction with Phusion High-Fidelity DNA Polymerase (New England Biolabs, Ipswich, MA, USA). Reaction conditions were 95 °C for 2 min, 33 cycles at 95 °C for 30 s, 50 °C for 15 s, and 72 °C for 1 min, and a final extension at 72 °C for 5 min. The PCR products were cloned into the pGem-T Easy vector (Promega, Madison, WI, USA) and transformed to One Shot TOP10 Chemically Competent *E. coli* (Thermo Fisher Scientific, Waltham, MA, USA), according to manufacturer instructions. Twelve randomly picked colonies were selected for plasmid purification and Sanger sequencing (Macrogen, Beotkkot-ro, Geumcheon-gu, Seoul). The resulting sequences were aligned to avoid allelic variation during sgRNA design and further high-resolution fragment analysis (HRFA) primer design. The Cas-Designer Tool (CRISRP RGEN Tools v1 www.rgenome.net/cas-designer, accessed on 10 July 2018) was used for sgRNA design, using one of the sequences obtained for *InvVac* as a query and *Solanum tuberosum* (PGSC v4.03) as a target genome [15]. The secondary structure for each sgRNA was analyzed by RNAFold software v1 (http://rna.tbi.univie.ac.at/cgi-bin/RNAWebSuite/RNAfold.cgi, accessed on 10 July 2018).

To edit lines for InvVac, four sgRNAs were designed: sgRNAG0, located in exon 1, and sgRNAG1, sgRNAG4, and sgRNAG10, also located in exon 1 (Table 1). These sgRNAs were cloned into the non-integrative pTRANS_100 vector under the *Arabidopsis thaliana* U6 promoter, following a Golden Gate-based protocol developed in Daniel Voyta’s lab [16]. This vector includes the coding sequence of the Cas9 nuclease protein under the control of the constitutive 35S promoter. We obtained two vectors with different combinations of sgRNA (Table 1), named crG0G4 and crG1G4. For multiplex editing, the RNPs were assembled in vitro by combining sgRNA157 (targeting the PPO2 gene) with sgRNAG0 (targeting the InvVac gene) and sgRNA157 (targeting the PPO2 gene) with sgRNAG10 (targeting the InvVac gene).

#### 2.1.2. Polyphenol Oxidase 2 Gene (PPO2)

We used the primers reported in González et al., 2020 [9] (Table 1) to amplify the *PPO2* gene from cv. Spunta. The PCR products were cloned and sequenced as described above to confirm the suitability of the previously designed sgRNAs (Table 1) for cv. Spunta.

### 2.2. Protoplast Transfection and Plant Regeneration

Protoplasts were isolated from 4-week-old plantlets according to González et al., 2020 [9]. To target *InvVac* in cv. Spunta, transfections were conducted by incubating 100,000 protoplasts with either crG0G4 or crG1G4 and a solution with 40% polyethylenglycol (PEG), 0.4 M mannitol, and 0.1 M Ca(NO_3_)_2_ for 30 min (experiment 1, E01). To target *InvVac* in cv. Atlantic, either 25% PEG or 40% PEG was employed in combination with the same vectors as above (experiment 2, E02). For the simultaneous targeting of *PPO2* and *InvVac* in cv. Spunta, we performed a transfection with ribonucleoproteins (RNPs) following the protocol described in [9]. We used the sgRNA157 [9] specific to the *PPO2* gene plus sgRNAG0 (experiment 3, E03) and sgRNAG10 specific to *InvVac* (experiment 4, E04). Regeneration controls were included for each cultivar, consisting of non-transfected protoplasts.

For plant regeneration, all protoplasts were embedded in sodium alginate and cultured for calli regeneration in Medium E, according to [9].

Green calli were released from alginate blobs after 21 days of culture and subcultured in Medium F until they reached a size of 2–3 mm. Full-grown calli were transferred to solid Medium H 30 days after transfection for shoot growth induction. To ensure the analysis of independent lines, several shoots were picked per callus and transferred to individual tubes with BM until root development. Samples from the leaves of the fully regenerated plantlets were picked for genomic DNA extraction and further analysis.

### 2.3. Identification of Edited Lines and Sequencing Analysis

Genomic DNA of the regenerated plants was extracted from leaves following the Haymes’s et al. (1996) [17] protocol.

#### 2.3.1. High-Resolution Fragment Analysis (HRFA)

The presence of mutations in the *InvVac* gene was determined by HRFA, according to [9]. Primer combinations HRFAG0R-FAM and InvVac-F1, HRFAG1R-VIC and InvVac-F4, and HRFAG4F-NED and InvVac-R5 were used for the analysis of the sgRNAG0, sgRNAG1, and sgRNAG4 target sites, respectively (Table 1). Primers were used to amplify a fragment of the target gene, using Phusion High-Fidelity DNA Polymerase (New England Biolabs). The reaction conditions were 98 °C for 1 min, 30 cycles of 98 °C for 30 s, 52 °C for 20 s, and 72 °C for 15 s, with a final extension of 72 °C for 5 min.

The labeled PCR products were analyzed in an Applied Biosystems 3500 Genetic Analyzer (Thermo Fisher Scientific, Waltham, MA, USA) (UGB sequencing service IABIMO Castelar, Buenos Aires, Argentina), using GeneScan 600 LIZ Dye Size Standard (Thermo Fisher Scientific, Waltham, MA, USA) as an internal lane size standard. Fragment length was determined with GeneMarker software v3.0.1 (SoftGenetics, www.softgenetics.com), and insertions or deletions were identified by comparing each line electropherogram versus the control.

#### 2.3.2. Detection of CRISPR-Induced Mutations

*InvVac* gene PCR amplification of the fragment of the selected edited lines from individual editions was performed by the Illumina MiSeq sequencing service (Genomic platform, Malbrán Institute, Buenos Aires, Argentina). Multiplex-edition *InvVac* and *PPO2* gene PCR amplification of the fragments of selected lines from individual editions was performed by the NGS sequencing service (Celemics, Republic of Korea). Sequencing data were analyzed using Geneious software version 2023.1.2 (https://www.geneious.com/), and insertions or deletions were identified by comparing each edited line to the wild-type control. Target gene fragments were amplified using the primers listed in Table 1, Q5 DNA Polymerase (New England Biolabs, Ipswich, MA, USA), and the following PCR conditions: initial denaturation at 98 °C for 5 min, 34 cycles of 98 °C for 30 s, 60 °C for 20 s, and 72 °C for 15 s, followed by a final extension at 72 °C for 5 min.

### 2.4. Plant Growth Conditions and Tuber Harvesting

Selected in vitro-regenerated plantlets were transferred to 3 L pots with soil and placed in a greenhouse under a 16:8 photoperiod. Fifteen biological replicates were grown for each edited line and for the non-edited control lines of cv. Spunta and cv. Atlantic. Tubers were harvested after 110 days of cultivation, just before plant senescence.

For the cold-induced sweetening resistance testing, tubers (both edited and non-edited) were divided into three groups:

Tubers stored at room temperature

Tubers stored at 4 °C for 15 days

Tubers stored at 4 °C for 60 days

After storage, a phenotypic evaluation of fried potato chips was conducted, along with the quantification of reducing sugars and sucrose.

For the bruising resistance testing, tubers (both edited and non-edited) were used for enzymatic browning assays and PPO activity measurements.

### 2.5. Fried Product Characterization

Potato slices were fried at 180 °C for 3 min or until bubbling ceased, then drained and placed on a white background for visual assessment. Chip color was scored using a nine-point reference chart, ranging from very light yellow (9) to very dark brown (1), developed by the Institute of Storage and Processing of Agricultural Products (Wageningen, Netherlands). Additionally, chip color was quantified with a Minolta CR-300 colorimeter, and luminosity (DW) was calculated based on the instrument’s L, a, and b values.

### 2.6. HPLC-Based Determination of Sucrose and Reducing Sugars

Five grams of frozen slices were weighed and homogenized in an ultraturrax at 11,000 rpm for 1 min with 20 mL of 80% ethanol (*v*/*v*). Sugars were extracted from the homogenate by incubation at 80 °C for 1 h. The homogenate was filtered and then centrifuged at 4 °C at 10,000× *g* for 10 min. The supernatant was eluted in a solid-phase extraction column, previously conditioned with methanol. Glucose and fructose concentration was determined by high-performance liquid chromatography (HPLC), a service provided by Fares Taie Laboratory, Mar del Plata, using an Amide-80 column, a mobile phase with 70% acetonitrile/water (*v*/*v*), and a flow rate of 1 mL/min, and by the Analyses of Chemical Residues Laboratory (LARQ-IPADS Balcarce, Argentina), using an ACQUITY BEH Amide column, a mobile phase with 75% acetonitrile/25% water/0.2% triethylamine (TEA), and a flow rate of 0.3 mL/min. Sugar quantification of the samples was carried out with external standards of glucose and fructose.

### 2.7. Enzymatic Browning and PPO Activity

Enzymatic browning and PPO activity for non-edited cv. Spunta and the edited line from transfections with RNPs for the *PPO2* gene were measured according to González et al. (2020) [10].

### 2.8. Field Trial of Line 6A

Line 6A, edited in the *InvVac* gene, was used to perform field assays in Río Primero, Córdoba province, Argentina. The trial was conducted with four plots of four rows each for the edited line 6A and for cv. Atlantic (control). The corresponding irrigation and phytosanitary treatments were applied. After 100 days, tubers were harvested, and a group of tubers of each line was stored at 4 °C for up to 120 days. Determinations of reducing sugar content by HPLC and fried product characterization were performed at harvest and at 30, 67, 74, 93, and 120 days post-storage at 4 °C, as described in the previous sections.

### 2.9. Statistical Analyses

Data were analyzed using a two-way ANOVA analysis. Multiple comparisons between treatments and lines were evaluated by Bonferroni’s test (*p* < 0.05). Regression analyses were performed using Sigmaplot 12.0 software [18].

## 3. Results

### 3.1. Identification of Edited Lines and Sequencing Analysis

#### 3.1.1. Single Editing for the Vacuolar Invertase Gene

A total of 76 and 70 potato lines were regenerated from protoplasts transfected with crG0G4 for cv. Spunta (E01) and cv. Atlantic (E02), respectively. Additionally, 114 lines were derived from cv. Spunta transfected with a new vector carrying a different sgRNA combination, crG1G4. Size differences in the amplified target regions were detected through HRFA, which revealed insertions and/or deletions in four lines (5.3% of the total analyzed) derived from cv. Spunta and seven lines (17% of the total analyzed) derived from cv. Atlantic. Based on the absence of the fragment size corresponding to the non-edited allele, lines designated 37S, 38S, and 75S (derived from cv. Spunta) and lines 6A, 13A, and 38A (derived from cv. Atlantic) contained mutations in all four alleles of the target gene. However, in-frame mutations were observed for some alleles in multiple lines (Table 2).

Amplicons from five lines lacking non-edited alleles at either target site were sequenced by NGS (Figure S1 and Table 2). Sequence analysis showed slight differences with the HRFA observations in some cases, particularly in 38S, 6A, and 38A (Figure S1 and Table 2). A single line derived from cv. Atlantic, line 6A, was identified as a full knockout, as it contained frameshift mutations in all alleles at the gRNAG0 target site (Table 2). NGS analysis confirmed that line 6A had two types of mutations in the target region of gRNA0: (i) a 2 bp deletion with a frequency of 76% of the total reads, and (ii) a 1 bp insertion with a frequency of 24% of the total reads, suggesting an allelic dosage of 3:1, respectively. In both cases, the translational reading frame was disrupted, which probably resulted in a loss of function of the target gene. The remaining lines showed wild-type alleles or in-frame mutations of at least one allele (Table 2).

#### 3.1.2. Multi-Target Editing for the Vacuolar Invertase and Polyphenol Oxidase 2 Genes

A total of 29 lines were obtained in the E03 experiment that combined sgRNA157 (PPO2 gene) with sgRNAG0 (InvVac gene), 23 of which were analyzed by NGS (Table 3). This analysis revealed that six lines (26%) contained mutations in the InvVac gene, while all 23 analyzed lines contained mutations in the PPO2 gene. In another experiment (E04), using a combination of sgRNA157 (PPO2 gene) with sgRNAG10 (InvVac gene), 119 lines were obtained, with 22 analyzed by NGS (Table 3). Mutations were identified in the InvVac gene in 14 lines (64%), while all 22 contained mutations in the PPO2 gene. The line E04-5B contained two edited alleles for the InvVac gene and three edited alleles for the PPO2 gene (Table 3), while line E03-3 was edited in all alleles of the InvVac gene and in two alleles of the PPO2 gene (Table 3). Edits consisted mainly of small deletions (Table 3). Notably, different allelic variants were found in some lines originating from the same callus, for instance, in E03-7A and 7B or E04-8A, 8B, and 8C (Table 3).

### 3.2. Tuber Production and Fried Product Characterization

**Figure 1:**
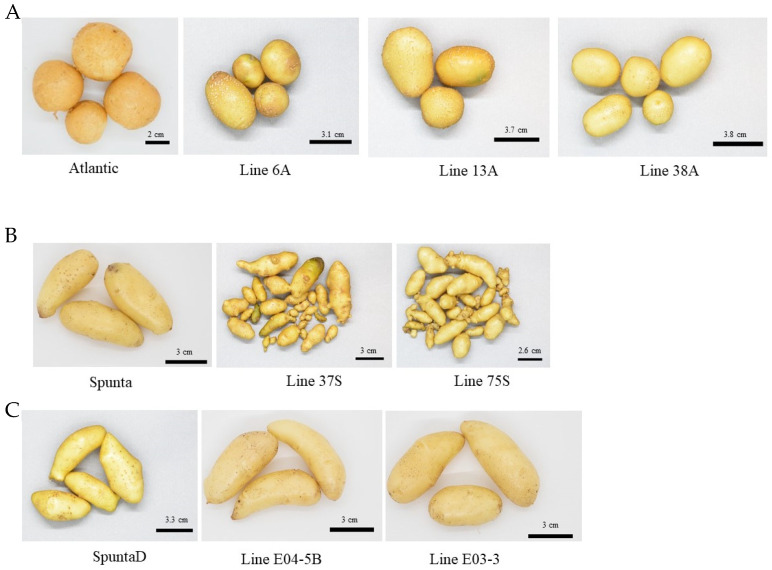
Tubers harvested from the greenhouse multiplication of each edited line and their respective control: (**A**) tubers from cv. Atlantic and edited lines 6A, 13A, and 38A; (**B**) tubers from cv. Spunta and edited lines 37 and 75; and (**C**) tubers from the double edited plants of cv. Spunta (non-edited control SpuntaD) and edited lines E04-5B and E03-3.

To assess resistance to cold-induced sweetening, 15 plants each from the 37S, 75S, 6A, 13A, 38A, E04-5B, and E03-3 edited lines were grown in a greenhouse, along with 15 plants each from cvs. Spunta and Atlantic as controls. The plants were cultivated from October to December, and the tubers were harvested 110 days after planting. Tubers from the edited cv. Atlantic lines (6A, 13A, and 38A) had a size and shape similar to those of the control (Figure 1A). In contrast, tubers from the edited cv. Spunta lines (37S and 75S) showed irregular shapes (Figure 1B). Tubers obtained from plants edited in both target genes exhibited a very similar phenotype to that of the control cv. Spunta (Figure 1C).

**Figure 2:**
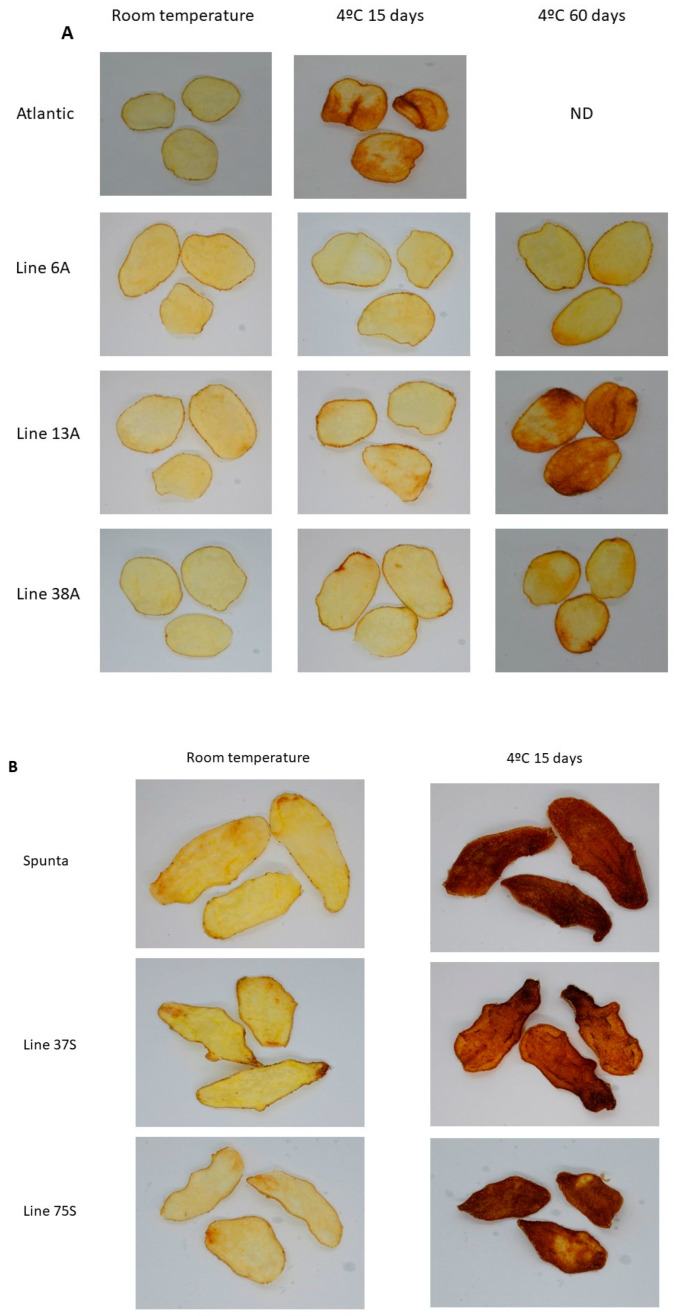
Fried product characterization. (**A**) Fried potato chips from non-edited (cv. Atlantic) and edited lines 6A, 3A, and 38A. (**B**) Fried potato chips from non-edited (cv. Spunta) and edited lines 37S and 75S.

The potato chip color was evaluated in tubers stored either at room temperature or at 4 °C for 15 and 60 days, respectively, using both a color reference chart and a colorimeter. The wild-type cv. Atlantic exhibited quality loss after 15 days at 4 °C, with an average color score of 4 (Figure 2A, Table 4) and a mean luminosity (DW) value of 52.1 (Table 4). In contrast, edited lines 6A, 13A, and 38A maintained acceptable potato chip quality after 15 days at 4 °C. The color card scores for lines 6A, 13A, and 38A were, on average, 8.3, 6.3, and 8, respectively (Figure 2B, Table 4), while the average DW values were 41.9, 47.3, and 45.2, respectively (Table 4). The wild-type cv. Spunta also exhibited reduced chip quality after 15 days at 4 °C, with an average color score of 1.3 (Figure 2B, Table 4) and a mean DW value of 59.9. Similarly, the edited lines 37S and 75S showed low color scores of 2 and 1.5, respectively (Figure 2B, Table 4). Their corresponding DW values (63.5 and 64.9) reflected increased dark coloration, as higher DW values are associated with a darker chip color (Table 4). Both methods indicated that line 6A exhibited the best performance after cold storage.

**Figure 3:**
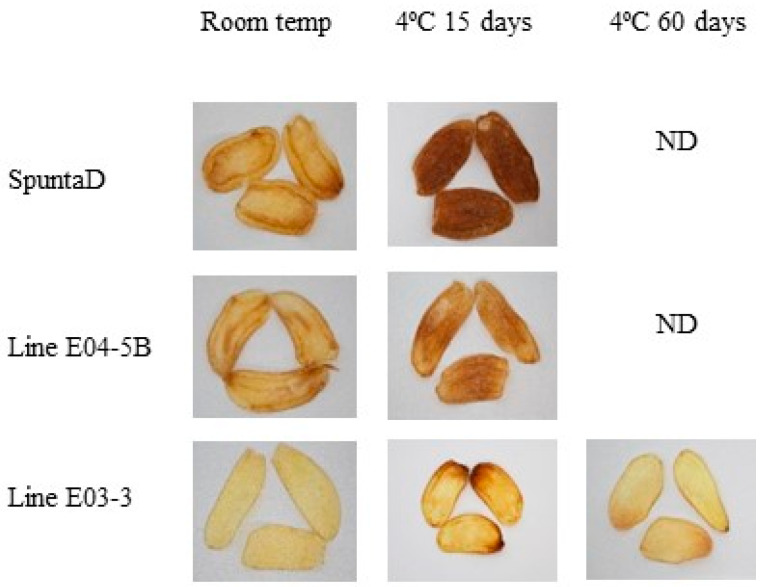
Fried product characterization from the double edited lines (E04-5B and E03-3) and their respective non-edited controls (cv. SpuntaD). ND: not determined.

**Figure 4:**
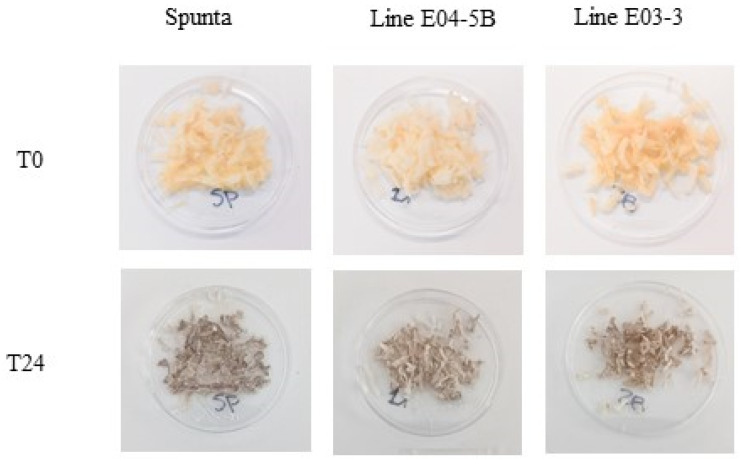
Discoloration of selected edited lines at 0 and 24 h after cutting. Tubers were randomly selected for each edited line and the non-edited control, grated raw, and exposed to the air for 24 h at room temperature (24 °C). Photos were taken immediately after grating (0 h) and 24 h later.

The non-edited SpuntaD lost potato chip quality after 15 days of storage at 4 °C (Figure 3), with an average color card score of 1.4 (Table 4) and an average DW of 61.1 (Table 4). Similarly, the edited line E04-5B presented a low color score (4) for potato chips (Figure 4, Table 4) and a DW value of 57.6, indicating a darker color (Table 4). Edited line E03-3 performed well after storage at 4 °C for 15 days, showing an average color card score of 7 (Figure 3, Table 4) and a DW value of 44.7 (Table 4). After 60 days, its quality decreased but to a lesser extent than the wild-type SpuntaD, with an average color card score of 5.3 (Figure 3, Table 4) and an average DW value of 58.2 (Table 4).

### 3.3. HPLC-Based Determination of Sucrose and Reducing Sugars

Reducing sugars and sucrose were quantified in raw potato chip slices using high-performance liquid chromatography (HPLC). Lines exhibiting higher chip color scores consistently showed lower levels of reducing sugars (Table 4). In contrast, lines with incomplete editing of all alleles showed an increase in sucrose concentration following cold storage (Table 4). Notably, the edited lines 6A, 38A, and E03-3 maintained reducing sugar levels below 3 mg/g of fresh weight after 60 days at 4 °C, showing statistically significant differences (*p* < 0.05) compared to their respective controls.

### 3.4. Enzymatic Browning and PPO Activity

**Figure 5:**
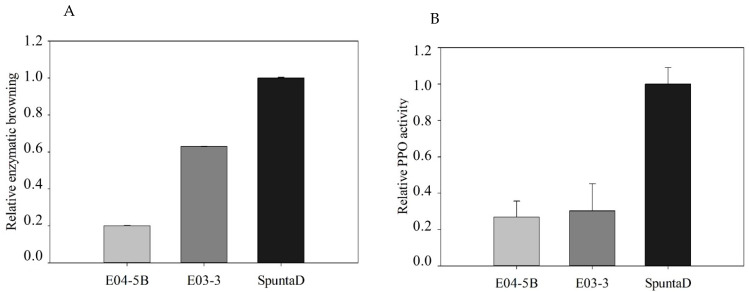
(**A**) Analysis of relative enzymatic browning and (**B**) relative PPO activity in tubers of the edited lines E03-3 and E04-5B. Each bar represents data from three technical replicates, each consisting of a sample with three biological replicates. Data are presented relative to the non-edited SpuntaD control line.

Phenotypic analysis of enzymatic browning and PPO activity was performed in E03-3, E04-5B, and the SpuntaD control. Initially, a qualitative analysis was conducted on tubers that were cut and exposed to air. Discoloration development was observed at 0 and 24 h after cutting (Figure 4). After 24 h of air exposure, the typical brown discoloration associated with oxidation was clearly visible in the SpuntaD control and, to a lesser extent, in lines E03-3 and E04-5B, with a slight difference in E04-5B (Figure 4). Enzymatic browning was quantitatively analyzed for each line and compared to the Spunta D control. Both edited lines exhibited reduced relative enzymatic browning compared to the control, with reductions of 80% in E04-5B and 40% in E03-3 (Figure 5A). A similar trend was observed for relative PPO activity, with E04-5B showing a 75% reduction and E03-3 a 70% reduction compared to SpuntaD (Figure 5B).

### 3.5. Field Trial of Line 6A Edited in the InvVac Gene

Line 6A, which showed the best performance among the edited lines for reduced cold-induced sweetening, was selected for the field trials. For registration purposes, it was renamed PIRU INTA and will be referred to as such in the manuscript from now on. Field trials conducted in Río Primero, Córdoba, Argentina—a region with moderate yielding potential—showed that PIRU INTA had a significantly lower yield than the Atlantic cultivar, yielding an estimate of 17 Tn/ha compared to 30 Tn/ha from Atlantic (a 56% reduction; *p* < 0.05).

**Figure 6:**
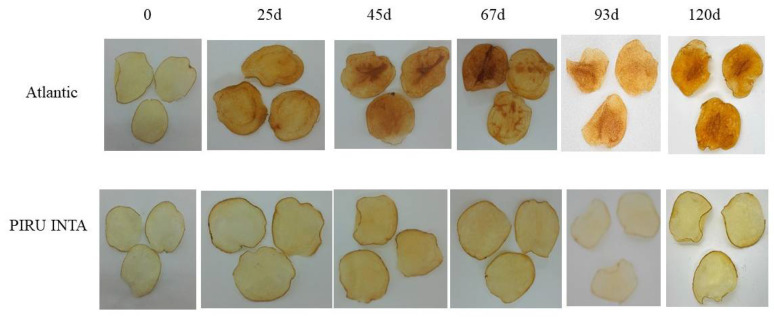
Fried product characterization for PIRU INTA and the cv. Atlantic control at different storage times at 4 °C.

**Figure 7:**
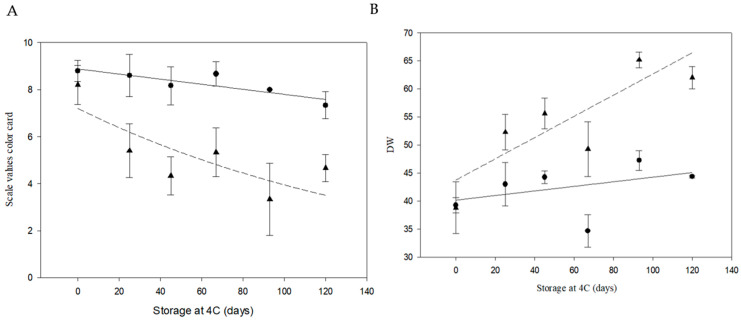
Phenotypic analyses of potato chip color from cv. Atlantic and PIRU INTA. (**A**) Color card developed by Institute of Storage and Processing of Agricultural Products in Wageningen, Netherlands, and a Minolta colorimeter. The color card has nine points, ranging from very light yellow (9) to very dark brown (1). Black circles represent PIRU INTA, and black triangles represent cv. Atlantic. The solid line represents the fit of PIRU INTA with R^2^ = 0.7424 and *p* = 0.013. The dashed line represents the fit of cv. Atlantic with R^2^ = 0.6190 and *p* = 0.035. (**B**) Minolta colorimeter. The values a, b, and L obtained from the colorimeter were used to calculate the DW parameter, which is an indicator of the whitening of the sample. Black circles represent PIRU INTA, and black triangles represent cv. Atlantic. The solid line represents the fit of PIRU INTA with R^2^ = 0.1505 and *p* = 0.3898. The dashed line represents the fit of cv. Atlantic with R^2^ = 0.6805 and *p* = 0.022.

Fried chip characterization was performed at harvest (no cold storage) and after storage at 4 °C (Figure 6). Quantification of reducing sugars was also performed on the same samples. A significant maintenance of quality was observed in PIRU INTA at 93 days post-storage (color card score average of 8.5) and at 120 days (color card score average of 7.3; Figure 7A). At all time points, no significant differences from time 0 were found in the color scores (*p* < 0.05). Additionally, significant differences (*p* < 0.05) were found between time 0 and days 25, 45, 67, and 93 after storage at 4 °C across all lines. Consistently, color analysis using a colorimeter showed that the luminosity values (DW, calculated from L, a, and b values) for days 25, 45, 67, 93, and 120 at 4 °C in cv. Atlantic exhibited significant differences (*p* < 0.05) compared to time 0. In contrast, for PIRU INTA, no significant differences were observed between storage times. Comparisons between PIRU INTA and non-edited cv. Atlantic showed significant differences at days 67 and 120 after storage at 4 °C (Figure 7B).

**Figure 8:**
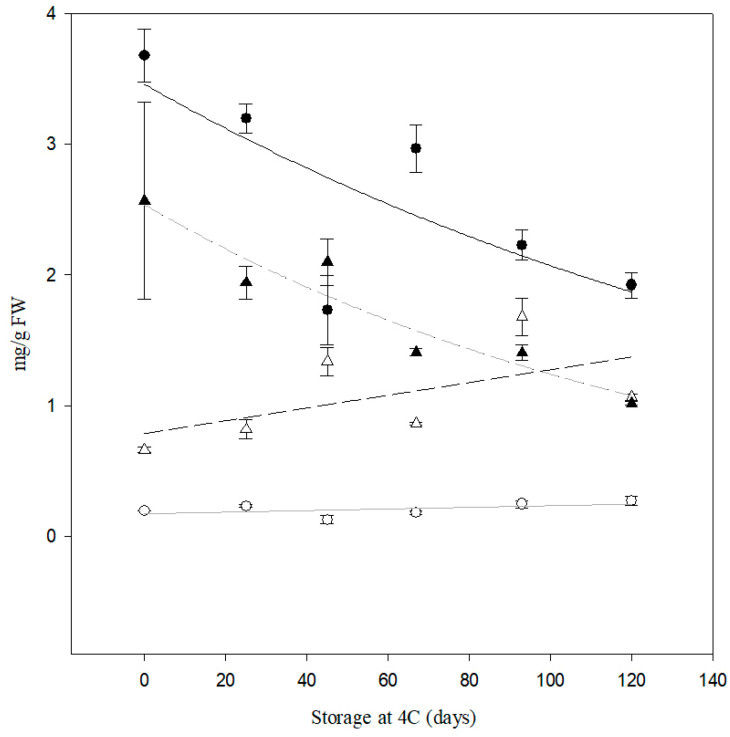
Quantification of reducing sugars (RS) and sucrose content by HPLC of tubers from cv. Atlantic and PIRU INTA from the field trial. Black circles represent the sucrose content of PIRU INTA, and black triangles represent the sucrose content of cv. Atlantic. White circles represent the RS content of PIRU INTA, and white triangles represent the RS content of cv. Atlantic. The black line represents the fit of sucrose PIRU INTA with R^2^ = 0.541 and *p* = 0.0956. The gray line represents the fit of RS PIRU INTA with R^2^ = 0.269 and *p* = 0.2916. The black dashed line represents the fit of sucrose cv. Atlantic with R^2^ = 0.912 and *p* = 0.003. The gray dotted line represents the fit of RS cv. Atlantic with R^2^ = 0.324 and *p* = 0.2382.

Reducing sugar content for PIRU INTA was significantly lower (*p* < 0.05) than for Atlantic for all the storage periods at 4 °C. Moreover, the differences in these values increased over time, with RS levels for PIRU INTA remaining unaltered (Figure 8). The sucrose content tended to decrease in both lines throughout the days of storage but showed great variability (Figure 8).

## 4. Discussion

The application of CRISPR/Cas9 technology in potato (*Solanum tuberosum* L.) breeding has enabled precise genome modifications to enhance post-harvest quality and storage potential. This study successfully developed gene-edited potato lines with improved resistance to CIS and enzymatic browning, two key factors affecting industrial processing and commercial value. Our findings further demonstrate that targeted gene editing can significantly enhance storage and processing quality in potato by addressing two quality-related traits simultaneously. The results reported here are in line with previous research showing that knockouts of vacuolar invertase (*InvVac*) and polyphenol oxidase 2 (*PPO2*) genes diminish quality deterioration in cold-stored and damaged tubers, respectively [4,5,6,7,8,9,10,11].

Cold storage is essential for maintaining tuber viability and reducing post-harvest losses; however, it promotes the conversion of sucrose into reducing sugars, negatively affecting fried product color and safety due to acrylamide formation [12]. In our study, we successfully edited the *InvVac* gene in four lines (5.3% of the total analyzed) derived from cv. Spunta and seven lines (17% of the total analyzed) derived from cv. Atlantic. All the lines obtained from cv. Spunta retained either unedited alleles or alleles containing in-frame mutations, which did not result in the desired CIS phenotype. After 15 days of storage at 4 °C, the frying quality of all Spunta-derived lines deteriorated drastically. In contrast, for cv. Atlantic, we obtained lines with all four *InvVac* alleles edited. Among them, only line 6A exhibited a complete knockout and maintained high-quality frying characteristics after cold storage. As observed in the cv. Spunta-derived lines, those lines containing alleles with in-frame mutations did not exhibit resistance to CIS. This is likely because of the presence of one or more alleles coding for a functional enzyme in these lines. Phenotypic analyses, including a chip color assessment and reducing sugar content measurements, confirmed the molecular findings that only lines 6A and 38A exhibited a favorable phenotype after storage for two months at 4 °C based on the absence of non-edited alleles for *InvVac* in those lines.

Additionally, we observed a direct correlation between optimal chip color (evaluated using a color card or colorimeter) and lower reducing sugar content (Figure 8). This aligns with the findings of [19], where reducing sugar levels were directly correlated with chip color. Line 6A, which exhibited full knockout of all alleles and the best CIS phenotype, was renamed as PIRU INTA and subjected to field trials for registration purposes. Our results demonstrated that PIRU INTA accumulated significantly fewer reducing sugars compared to its wild-type counterpart, cv. Atlantic. After 120 days at 4 °C, PIRU INTA retained an optimal color card score of 8.5, whereas cv. Atlantic showed a significant quality decline, with a score of 4 as early as 40 days of storage. These findings are consistent with prior research showing that *InvVac* knockout lines maintain lower reducing sugar levels, improving frying quality and reducing acrylamide formation [11,12,13]. Furthermore, the sustained low reducing sugar content for up to 120 days post-harvest highlights the potential for extended storage without compromising industrial processing standards.

Unlike the findings reported by Bhaskar et al. (2010) and Yasmeen et al. (2022) [4,5,6,7,8,9,10,11,12,13,14,15,16,17,18,19,20], PIRU INTA exhibited a 56% reduction in yield in the Córdoba trial. This outcome may be related to the central role of vacuolar invertase in carbohydrate metabolism [21], as it influences sugar accumulation, regulates carbohydrate composition in tubers, and affects the distribution of sucrose and hexoses. Despite this potential yield penalty, PIRU INTA offers other advantages in terms of post-harvest quality, maintaining chip color for extended periods of cold storage, an essential trait for industrial processing. This improved storage performance could translate into tangible economic and logistic benefits. In south-east Buenos Aires, the main potato production area of Argentina, for example, local potato stocks are often exhausted by April–May, forcing processing industries to source tubers from distant lower yielding locations such as Córdoba and Tucumán (approximately 800 km and 1100 km, respectively, from the factories’ location), thereby increasing transportation costs. Concurrently to lower yields, the cost per ton of the products of these regions is also higher. The availability of a locally grown variety like PIRU INTA with enhanced storage and processing qualities could reduce dependence on long-distance sourcing and improve overall supply chain efficiency. Moreover, late sowings and/or early frost occurrence may alter the quality of unharvested potatoes with soil temperatures under 4 °C, a damage that can be avoided using the improved variety. The recent results of a new trial in Balcarce—one of the highest potato yielding areas of Argentina, with a mean yield of 45 Tn/ha—rendered 52 Tn/ha of PIRU INTA compared to 72 Tn/ha of Atlantic, suggesting that the yield penalty of knocking down the *InvVac* gene can be minimized to acceptable levels.

PPO2 plays a critical role in enzymatic browning by catalyzing the oxidation of polyphenols to quinones, which subsequently polymerize into dark pigments [3]. A multiplex-editing approach targeting both *InvVac* and *PPO2* provides an effective strategy for developing potato varieties with improvements in these two crucial post-harvest traits. Our research group previously obtained full *PPO2* knockout lines in cv. Desirée, which showed a 73% reduction in enzymatic activity and a 63% decrease in enzymatic browning. In the present study, we aimed to generate cv. Spunta lines with edits in both *InvVac* and *PPO2*. We obtained two edited lines, E03-3 and E04-5B, with two and three edited alleles, respectively, resulting in reduced enzymatic browning. This was qualitatively confirmed by lower discoloration scores. Furthermore, a quantitative measurement of enzymatic browning in tubers showed reductions in lines E03-3 and E04-5B of 40% and 80% compared to the control, respectively. Lower levels of browning in the tubers of these lines coincide with the observed reductions in PPO activity levels. Thus, reductions of 70% and 74% compared to the control were determined for PPO activity in lines E03-3 and E04-5B, respectively. Despite the presence of the remaining non-edited allele/s in both lines, the induced edits in *PPO2* caused significant reductions in the total enzymatic activity in the tubers. This observation is in line with previous studies in potato, which have reported that the induction of mutations in multiple alleles can significantly alter gene function despite the presence of one or more predictively active alleles [9,10,11,12,13,14,15,16,17,18,19,20,21,22]. Wszelaczyńska et al., 2007, [23] reported a strong correlation between visual and absorbance-based methods. Conversely, in our study, visual tuber discoloration did not fully reflect the quantified reductions in enzymatic activity and browning. In addition to the reduced enzymatic browning, line E03-3 demonstrated resistance to CIS, evidenced by a lower reducing sugar content after 60 days of storage at 4 °C, confirming the stacking of both quality traits in a single line. We did not obtain any lines with all four alleles edited for both genes, likely due to the slightly lower efficiency of multiplex editing compared to single-gene editing. This suggests that further optimization of multiplex-editing strategies could enhance both traits simultaneously. Moreover, subsequent editing experiments on lines E03-3 and E04-5B could render full allele knockout on both genes.

Traditional potato breeding for improved storage and processing traits is a slow and complex process due to the crop’s tetraploid genome and high heterozygosity [8]. In contrast, CRISPR/Cas9 technology allows precise, targeted modifications with high specificity, reducing the need for extensive backcrossing and selection cycles. Our approach significantly accelerated the development of storage-resistant potato lines compared to conventional breeding methods, demonstrating the efficiency of genome editing in addressing post-harvest challenges [11]. Moreover, gene editing techniques allow the improvement of the existing successful varieties in key traits, maintaining the advantage of the previous allelic combination of the rest of the genome [7]. Additionally, the non-transgenic nature of our edited lines aligns with regulatory frameworks that favor genome-edited crops without foreign DNA insertion, enhancing their commercial acceptability [24,25].

The improved resistance to CIS and enzymatic browning in our edited potato lines offers substantial benefits to the food industry. Reduced sugar accumulation translates into lower acrylamide levels, mitigating health risks associated with fried potato consumption [26]. Additionally, the enhanced cold storage stability of PIRU INTA, without significant quality deterioration, reduces storage losses, contributing to a more efficient supply chain and lower environmental impact. A genome-editing strategy that does not compromise yield in CIS-resistant potatoes would be desirable. For example, CRISPR/Cas9-mediated editing of the *InvVac* intron 2 enhancer significantly reduced *InvVac* expression under cold storage conditions, confirming its cold-responsive function in cv. Katahdin [27]. Other strategies targeting genetic factors involved in the expression on *InvVac* triggered by cold may also provide an alternative approach.

From an economic standpoint, reducing post-harvest losses significantly enhances profitability for potato processors by minimizing waste and decreasing dependence on costly anti-sprouting agents such as chlorpropham (CIPC), which has been restricted in some markets due to environmental and health concerns [28]. The ability to store potatoes at low temperatures without compromising chip quality also contributes to lower refrigeration costs while preserving tuber viability.

Moreover, environmental conditions, particularly extreme temperatures driven by climate change, may expose potato crops to chilling conditions before harvest [29]. CIS-resistant varieties could help minimize reducing sugar accumulation in unharvested tubers.

The use of high-quality tubers with fewer processing defects reduces the need for surplus raw materials, thereby lowering the overall water footprint associated with potato production. This is particularly relevant given the water-intensive nature of potato processing, which includes multiple stages such as washing, peeling, and frying [30]. By enhancing processing efficiency and reducing tuber rejection due to poor quality, our gene-edited lines contribute to more sustainable and resource-efficient agricultural practices.

## 5. Conclusions

Our study highlights the potential of CRISPR/Cas9-mediated genome editing in developing potato varieties with enhanced cold storage resistance and improved processing quality. The successful reduction of CIS and enzymatic browning has direct implications for food safety, industry efficiency, and sustainability. By lowering acrylamide formation, reducing food waste, and optimizing supply chain efficiency, these advancements contribute to a healthier, more sustainable, and economically viable potato industry. Further research should explore large-scale field trials and consumer acceptance studies to facilitate the widespread adoption of these improved potato lines.

## Figures and Tables

**Table 1:** Primers and sgRNA guides used in this study.

Primer Name	Sequence (5’–3’)	Purpose
InvVac-F1	CAATTCAGTTGCCCCCTGTC	Sequence analysis of *InvVac* gene of *Solanum tubersoum* cv. Spunta and cv. Atlantic.
InvVac-R5	CGCACGATTATTGTGTATGGTGCA	
sgRNAG0	CCTCCCATTACACATTCCTC	sgRNA guide for *InvVac.*
sgRNAG1	CTATTTGGGGAAATATCACA	sgRNA guide for *InvVac.*
sgRNAG4	GAAGAAACAACGAAGAGTAC	sgRNA guide for *InvVac.*
sgRNAG10	GGTCAAGTACAAAGGCAACC	sgRNA guide for *InvVac.*
sgRNA157	TTTTCGATGTAACACGTGAC	sgRNA guide for *PPO2* from González et al., 2020 [9].
HRFAG0R-FAM	TCGGAAAGAAGGCTACAGAAAG	Amplification of *InvVac* gene fragment spanning the sgRNAG0 target site for HRFA and NGS. This primer was combined with InvVac-F1.
HRFAG4F-NED	TGGGTTGAAGCTGGATTATGG	Amplification of *InvVac* gene fragment spanning the sgRNAG4 target site for HRFA. This primer was combined with InvVac-R5.
HRFAG1R-VIC	ATCGTACCATTGATCAGGAACC	Amplification of *InvVac* gene fragment spanning the sgRNAG1 target site for HRFA.
InvVac-F4	TTGGTCAACAGGTCCATTGT	
PPO2_2Bf	GCTCCATTTCGGTGACTTT	Amplification of *PPO2* gene fragment spanning the sgRNA157 target site for NGS from González et al., 2020 [9].
PPO2_2Br	TGGTGGCAAAGAGTTACAAG	
G2-R	TGGTTCCTGATCAATGGTAC	Amplification of *InvVac* gene fragment spanning the sgRNAG10 target site for NGS.
G3-R	GTCCAAGCAGTGGTGGGGTC	

**Table 2:** Edited lines detected by HRFA and NGS. G0 and G4 are the sgRNA guides specific to the *InvVac* gene. (+) indicates nucleotide insertions, and (−) indicates nucleotide deletions. ND: not determined.

Cultivar	Line	Allelic Variants in Target Site sgRNAG0 by HRFA	Allelic Variants in Target Site sgRNAG4 by HRFA	Allelic Variants in Target Site sgRNAG0 by NGS	Allelic Variants in Target Site sgRNAG4 by NGS
Spunta	37S	−2; −5; −6; −12	0	−2; −5; −6; −12	0
	38S	−3	−2; −3; −5	ND	−2; −3; −5
	44S	−2; 0	0	ND	ND
	75S	−1; −3; −7	0	−1; −3; −7	0
Atlantic	6A	+2; −2; −4	0	+1; −2	0
	13A	−3; −4	−6; −12; −28; 0	ND	ND
	16A	0; −3; −6	0	ND	ND
	30A	0; −3	0	ND	ND
	38A	+1; −1	0	+1; −1; 0	0

**Table 3:** Edited lines per experiment (E03 and E04) detected by NGS. G0 and G10 are the sgRNA guides specific to the *InvVac* gene, and G157 is the sg RNA guide specific to the PPO2 gene. (+) indicates nucleotide insertions, and (−) indicates nucleotide deletions. NR: no results obtained.

Line	Allelic Variants G0	Allelic Variants G157	Line	Allelic Variants G10	Allelic Variants G157
E03-2A	−1; 0	−2; 0	E04-2A	−1; −1; 0	−2; 0
E03-3	+3; −5; −4; −2	−2; –1; 0	E04-2B	0	−1; −1; 0
E03-4	0	2; 0	E04-3	−1; 0	NR
E03-5A	0	−2; 0	E04-4A	−1; −1; 0	−2; 0
E03-5B	0	−2; 0	E04-4B	−1; −1; 0	−2; 0
E03-6A	−2; −1; 0	−2; 0	E04-4C	−1; 0	NR
E03-7A	0	−2; 0	E04-4D	0	−2; −1; 0
E03-7B	−1; 0	−2; 0	E04-5A	+1; −1; 0	+1; −1; −2; 0
E03-8A	NR	−2; 0	E04-5B	+1; −1; 0	+1; −1; −2; 0
E03-8D	0	−2; 0	E04-5C	+1; −1; 0	+1; −1; −2; 0
E03-10B	NR	−2; 0	E04-5D	+1; −1; 0	NR
E03-11C	0	−2; −47; −49; 0	E04-6A	0	−2; 0
E03-14B	0	−2; −49; −47; 0	E04-6B	−2; 0	−2; 0
E03-15C	−1; 0	−2; −35; −49; 0	E04-6C	0	−2; 0
E03-17B	0	−2; −14; 0	E04-6D	−1; 0	−2; −1; 0
E03-17D	0	−2; 0	E04-6E	0	−2; 0
E03-20B	−1; −2; 0	−2; 0	E04-6F	−1; 0	−2; 0
E03-20C	0	−2; −1; 0	E04-7A	0	−2; 0
E03-21B	0	−2; −5; 0	E04-7B	−1; 0	−2; −1; 0
E03-28	0	−2; 0	E04-8A	0	−2; −1; 0
E03-29	0	−2; 0	E04-8B	0	−1; 0
E03-33B	0	NR	E04-8C	−1; 0	−2; −1; 0
E03-34	0	−2; 0			

**Table 4:** Phenotypic characterization of the edited lines and their respective controls. The non-edited control for multiplex editing was designated as “SpuntaD” to differentiate it from the control Spunta used for lines targeted solely in the VacInv gene. Different capital letters determine significant differences (*p* < 0.05) between different conditions within the same line. ND: not determined.

Line	Storage at 4 °C (Days)	Average Scale Values of Color Card	Average DW	Average Reducing Sugar (mg/gr. FW)	Average Sucrose (mg/gr. FW)
Atlantic	0	8.8 ± 0.45 A	36.6 ± 3.4 A	0.6 ± 0.02 A	2.6 ± 0.75 A
	15	4 ± 0 B	52.1 ± 3.5 B	7.2 ± 0.69 B	6.1 ± 0.46 B
6A	0	8.3 ± 0.5 A	44.6 ± 1.7 A	0.4 ± 0.01 A	7.7 ± 1.75 A
	15	8.3 ± 0.96 A	41.9 ± 3.5 A	0.7 ± 0.18 A	3.5 ± 0.27 B
	60	8 ± 0.71 A	42.1 ± 4.9 A	0.9 ± 0.03 A	7.8 ± 1.39 A
13A	0	8.3 ± 0.96 A	41.7 ± 2.7 A	0.2 ± 0.07 A	1.8 ± 1.13 A
	15	6.3 ± 0.5 B	47.3 ± 4.3 A	1.8 ± 0.61 B	4.7 ± 1.45 A
	60	3 ± 0 C	54.7 ± 4.7 B	6.3 ± 1.33 C	3.0 ± 0.59 A
38A	0	8.8 ± 0.5 A	36.2 ± 1.7 A	0.1 ± 0.02 A	1.1 ± 0.10 A
	15	8 ± 0.82 A	45.2 ± 4.3 B	0.8 ± 0.27 A	5.5 ± 2.14 B
	60	6.4 ± 0.55 B	47.3 ± 1.3 B	2.4 ± 0.52 B	5.5 ± 2.20 B
Spunta	0	7.8 ± 1.5 A	45.1 ± 4.0 A	0.7 ± 0.04 A	2.9 ± 0.87 A
	15	1.3 ± 0.5 B	59.9 ± 2.2 B	7.9 ± 0.58 B	2.2 ± 0.31 A
37S	0	7.3 ± 1.5 A	47.9 ± 0.8 A	2.7 ± 0.64 A	2.7 ± 1.32 A
	15	2 ± 1.41 B	63.5 ± 1.8 B	6.8 ± 1.61 A	7.7 ± 1.76 B
75S	0	7.3 ± 1.5 A	42.7 ± 6.4 A	0.9 ± 0.05 A	2.2 ± 0.42 A
	15	1.5 ± 1 B	64.9 ± 0.2 B	8.6 ± 0.55 A	6.8 ± 0.20 B
SpuntaD	0	4.8 ± 0.4 A	53.5 ± 1.84 A	2.3 ± 0.26 A	1.5 ± 0.55 A
	15	1.4 ± 0.5 B	61.1 ± 0.96 B	10 ± 2.61 A	1.7 ± 0.15 A
E04-5B	0	6 ± 0 A	45.9 ± 1.56 A	ND	ND
	15	4 ± 0.7 B	57.6 ± 2.14 B	ND	ND
E03-3	0	9 ± 0 A	41.6 ± 3.97 A	0.4 ± 0.03 A	1.7 ± 0.36 A
	15	7 ± 0 B	44.7 ± 2.32 A	1.5 ± 0.89 A	7.1 ± 0.03 B
	60	5.3 ± 0.6 C	58.1 ± 0.68 B	2.9 ± 0.13 B	9.1 ± 0.21 B

## Data Availability

The original contributions presented in this study are included in the article/Supplementary Material. Further inquiries can be directed to the corresponding author(s).

## Supplementary Materials

The following supporting information can be downloaded at: https://www.mdpi.com/article/10.3390/biology14040445/s1, Figure S1: (**A**) Sequenced alleles of *InvVac* gene in lines 6A, 38A, 37S, 38S, 75S and wild type sgRNAG0 region. (**B**) Sequenced alleles of *InvVac* gene in line 38S and wild type sgRNAG4 region. Red letters indicate insertions or deletions.

## Abbreviations

The following abbreviations are used in this manuscript:

CRISPR/Cas9	Clustered Regularly Interspaced Short Palindromic Repeats/CRISPR-associated nuclease 9
GMO	Genetically modified organisms
RS	Reducing sugar
cv.	Cultivar
CIS	Cold-induced sweetening
PPO2	Polyphenol oxidase 2
InvVac	Vacuolar invertase
sgRNA	Single guide RNA
HPLC	High-performance liquid chromatography
HRFA	High-resolution fragment analysis
NGS	Next-generation sequencing
RNP	Ribonucleoprotein complex
