# Correction: Massa et al. CRISPR/Cas9-Mediated Development of Potato Varieties with Long-Term Cold Storage and Bruising Resistance. *Biology* 2025, *14*, 445

**DOI:** 10.3390/biology14070768

**Published:** 2025-06-26

**Authors:** Gabriela Alejandra Massa, Cecilia Andrea Décima Oneto, Matías Nicolás González, Anabela Poulsen Hornum, Ailín Arizmendi, Sofía Sucar, Silvina Beatriz Divito, Sergio Enrique Feingold

**Affiliations:** 1 Laboratorio de Agrobiotecnología, EEA Balcarce-IPADS (UEDD INTA–CONICET), Instituto Nacional de Tecnología Agropecuaria (INTA), Balcarce B7620, Argentina; cdecimaoneto001@dundee.ac.uk (C.A.D.O.); matias.gonzalez@slu.se (M.N.G.); poulsenhornum.a@inta.gob.ar (A.P.H.); arizmendi.ailin@inta.gob.ar (A.A.); sofiasucar@yahoo.com.ar (S.S.); divito.silvina@inta.gob.ar (S.B.D.); 2 Facultad de Ciencias Agrarias, Universidad Nacional de Mar del Plata, Balcarce B7620, Argentina

**Error in Figure**

In the original publication [1], there was a mistake in Figure 6 as published. During the editing process, the images corresponding to potato chips stored at 4 °C for 45 days for both Atlantic and PIRU INTA were inadvertently replaced with the images for 25 days, resulting in duplicated images for both time points. The corrected Figure 6, which now includes the appropriate image for 45 days of storage, appears below. The authors state that the scientific conclusions are unaffected. This correction was approved by the Academic Editor. The original publication has also been updated.

**Text Error in the Acknowledgements:**

The first sentence “We want to thank David Douches for welcoming me into his laboratory for four months” has been changed to: “We want to thank David Douches for welcoming G. Massa into his laboratory for four months”.

The authors state that the scientific conclusions are unaffected. This correction was approved by the Academic Editor. The original publication has also been updated.

## Figures and Tables

**Figure 6:**
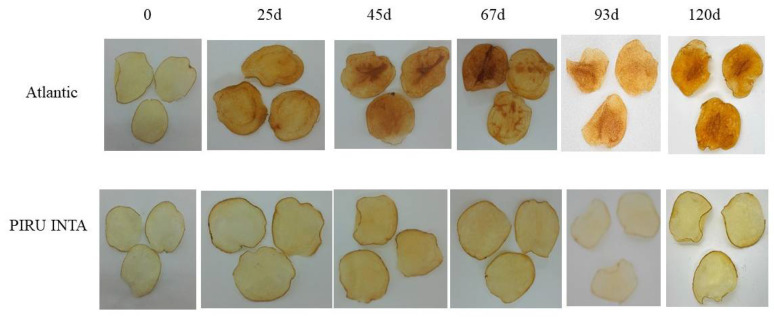
Fried product characterization for PIRU INTA and the cv. Atlantic control at different storage times at 4 °C.
