# Supplementary material for: CRISPR/Cas9-Mediated Development of Potato Varieties with Long-Term Cold Storage and Bruising Resistance
Source: Biology (Basel). 2025 Apr 20;14(4):445. doi: 10.3390/biology14040445 (PMC12024581; doi:10.3390/biology14040445)
Supplement: Supplementary file 1 [file biology-14-00445-s001.zip › biology-3595226-supplementary.pdf]

A

Sequence sgRNAG0 in cv. Atlantic and cv. Spunta	CCGCCTCCCATTACACATTCCTCCCGGA
Allele 1-6A (-2)	CCGCCTC--ATTACACATTCCTCCCGGA
Allele 2-6A (+1)	CCGCCTCCCCATTACACATTCCTCCCGGA
Allele 1-38A (-1)	CCGCCTCC--ATTACACATTCCTCCCGGA
Allele 2-38A (+1)	CCGCCTCCCCATTACACATTCCTCCCGGA
Allele 3-38A (+1)	CCGCCTTCCCATTACACATTCCTCCCGGA
Allele 1-37S (-2)	CCGCCT--CATTACACATTCCTCCCGGA
Allele 2-37S (-5)	CCGCC-----TTACACATTCCTCCCGGA
Allele 3-37S (-6)	CCGCCT------ACACATTCCTCCCGGA
Allele 4-37S (-12)	CCGCC------------TTCCTCCCGGA
Allele 1-75S (-1)	CCGCCTCC-ATTACACATTCCTCCCGGA
Allele 2-75S (-3)	CCGCCT---ATTACACATTCCTCCCGGA
Allele 3-75S (-3)	CCGCC---CATTACACATTCCTCCCGGA
Allele 4-75S (-7)	CCGCCT-------CACATTCCTCCCGGA

B

Sequence sgRNAG4 in cv. Spunta	CCCGAAGAAACAACGAAGAGTAC
Allele 1-38S (-2)	CCCGAA--AACAACGAAGAGTAC
Allele 2-38S (-3)	CCCGAA---ACAACGAAGAGTAC
Allele 3-38S (-5)	CCCGAA-----AACGAAGAGTAC

Figure S1: (**A**) Sequenced alleles of *InvVac* gene in lines 6A, 38A, 37S, 38S, 75S and wild type sgRNAG0 region. (**B**) Sequenced alleles of *InvVac* gene in line 38S and wild type sgRNAG4 region. Red letters indicate insertions or deletions.
